# International Consensus on the Diagnosis and Clinical Management of Idiopathic Short Stature: A Modified Delphi Method

**DOI:** 10.1159/hrp/adaag008

**Published:** 2026-08-27

**Authors:** Agnès Linglart, Stefano Cianfarani, Mehul T. Dattani, Alexander A.L. Jorge, Yael Lebenthal, Xiaoping Luo, Mohamad Maghnie, Bradley S. Miller, Haruo Mizuno, Robert Rapaport, Paulo Ferrez Collett-Solberg, Andrew Dauber

**Affiliations:** a Paris Saclay University, AP-HP Endocrinology and Diabetes for Children, Bicêtre Paris-Saclay Hospital, le Kremlin-Bicêtre, France; b Department of Systems Medicine, University of Rome ‘Tor Vergata’, Rome, Italy; c Endocrinology and Diabetes Unit, Bambino Gesù Children’s Hospital, IRCCS, Rome, Italy; d Department of Women's and Children's Health, Karolinska Institute, Stockholm, Sweden; e Genetics and Genomic Medicine Research and Teaching Department, University College London, Great Ormond Street Hospital Institute of Child Health, London, UK; f Genetic Endocrinology Unit (LIM25), Endocrinology Division, Hospital das Clínicas da Faculdade de Medicina da Universidade de São Paulo (HC-FMUSP), São Paulo, Brazil; g The Institute of Pediatric Endocrinology, Diabetes & Metabolism, Dana-Dwek Children's Hospital, Tel Aviv, Gray Faculty of Medical & Health Sciences, Tel Aviv University, Tel Aviv, Israel; h Department of Pediatrics, Tongji Hospital, Tongji Medical College, Huazhong University of Science and Technology, Wuhan, China; i Department of Neuroscience, Rehabilitation, Ophthalmology, Genetics, Maternal and Child Health, University of Genoa, Genoa, Italy; j Pediatric Endocrinology Unit, IRCCS Istituto Giannina Gaslini Genoa, Italy; k University of Minnesota Medical School, Minneapolis, MN, USA; l Fujita Health University School of Medicine, Toyoake City, Japan; m Icahn School of Medicine at Mount Sinai, New York, NY, USA; n Discipline of Endocrinology, Faculdade de Ciências Médicas da Universidade do Estado do Rio de Janeiro (UERJ), Rio de Janeiro, Brazil; o Division of Endocrinology, Children’s National Hospital and Department of Pediatrics, The George Washington University School of Medicine and Health Sciences, Washington, DC, USA

**Keywords:** idiopathic short stature, diagnosis, Delphi method, consensus, management

## Abstract

**Introduction:**

Idiopathic short stature (ISS) refers to children whose height falls substantially below the mean for sex and age relative to a reference population, despite the absence of an identifiable endocrine, genetic, syndromic, or systemic disorder. Clinical management remains challenging because no dedicated ICD-11 code exists and no universally accepted diagnostic criteria have been established. This has led to variability in how the condition is classified, inconsistency in access to testing, and uncertainty around eligibility for growth-promoting therapies. To address these inconsistencies and support broader global alignment in the recognition and management of ISS, an international consensus initiative was undertaken.

**Methods:**

A Steering Committee of 10 internationally recognized experts with extensive clinical and academic expertise in ISS supported the development of a structured modified Delphi process. An Expert Panel of 22 key opinion leaders participated in a three-round survey conducted between November 2025 and February 2026. Panelists evaluated draft statements related to ISS definition, diagnostic criteria, burden of disease, and decision to treat. Consensus thresholds were predefined at 70%, and statements were refined between rounds, as needed.

**Results:**

A total of 44 statements achieved consensus, with final agreement levels ranging from 77% to 100%. The statements establish a standardized clinical definition of ISS, propose evidence-based diagnostic criteria applicable across diverse healthcare settings, describe the impact of ISS on patients and caregivers, and suggest when to consider growth-promoting treatment for patients with ISS. For example, consensus was reached on the definition of ISS (a condition in which an individual has a height more than a standard deviation score of 2 below the mean for sex, age, and population, corresponding to below the 2.3 percentile, without identifiable underlying pathology) and on key elements of diagnostic evaluation (include the child's medical, developmental, and medication background, including prenatal and perinatal course, chronic illnesses or infections, and neurodevelopmental milestones, alongside family history and environmental exposures; perform a physical examination to identify clinical signs of underlying pathology, including assessment of body proportions, dysmorphic body features, head circumference, body mass index, and pubertal development). Key areas requiring future research were also highlighted.

**Conclusion:**

This international consensus offers the first globally derived, ISS-focused guidance developed using a formal Delphi methodology for identifying and managing children and adolescents with ISS. Adoption of these recommendations may reduce regional variability, improve diagnostic clarity, and ultimately enhance outcomes for individuals affected by ISS.

## Introduction

Idiopathic short stature (ISS) describes children whose height falls substantially and persistently below the mean for sex and age relative to a reference population, despite the absence of any identifiable disorder affecting the growth hormone (GH)/insulin-like growth factor (IGF) axis or evidence of an endocrine, genetic, syndromic, or organ system disorder. ISS is a diagnosis of exclusion and may therefore evolve over time as additional clinical features emerge, or as more advanced diagnostic testing becomes available [1].

Globally, by definition, approximately 2–3% of children meet criteria for short stature [2], and an estimated 60–80% of these cases are idiopathic [3, 4]. However, there remains a lack of consensus within the pediatric endocrinology community regarding diagnostic criteria for ISS. Challenges in the diagnosis and treatment of ISS stem from the lack of universally accepted diagnostic criteria, leading to variability in classification and inconsistencies in access and reimbursement. These issues are further compounded by regional variation in the availability of genetic testing and whether ISS is an approved indication for genetic investigation and GH treatment. This creates challenges for children and caregivers, leading to possible underestimation of the burden of short stature and, ultimately, variations in the care offered to many children with ISS. Delayed or missed diagnosis of ISS can also prolong psychosocial burden, as children with untreated short stature may exhibit higher internalizing symptoms and poorer health-related quality of life (HRQoL) compared with peers who reach normal height [5]. Furthermore, postponing diagnosis and treatment may narrow the window to achieve improvements in height and HRQoL.

Multiple regional consensus efforts have contributed valuable insights and highlighted the need to update the 2008 international consensus statement. However, their variability, including differing thresholds for short stature (standard deviation scores [SDS] of 2, 2.25, or 2.5 below mean), diagnostic tests, and treatment eligibility, underscores the broader issue of global misalignment in the diagnostic evaluation of ISS [2, 6–8]. To promote consistency in clinical practice and support the broader global recognition of ISS, a modified Delphi process was undertaken, with the resulting consensus statements focused specifically on the clinical diagnosis of ISS. The process used iterative survey rounds. Round 1 was non-anonymized, Round 2 was conducted in a single-blind format, with responses visible only to the steering committee and facilitator, and Round 3 involved circulation of the revised statements for final consensus. Given the relatively limited field of experts for this condition, full anonymization was not deemed necessary.

This modified Delphi process brought together international experts to address these gaps and establish a unified, globally relevant clinical diagnostic framework for ISS that will set the grounds for future management and treatment guidelines. Here, we report the international diagnostic consensus statements for ISS, which were developed to reflect agreement among global experts and support harmonized clinical practice. Guidance related to genetic evaluation, which is beyond the scope of the current workstream, has been provided in the recently published International Guidelines on Genetic Testing of Children with Short Stature [9]. The primary objectives of this consensus effort were to: (1) establish a standardized clinical definition of ISS; (2) present statements on the burden of ISS on children and caregivers; (3) develop evidence-based diagnostic criteria applicable across diverse healthcare settings; (4) provide guidance on when to consider growth-promoting treatment; and (5) identify key areas requiring future research.

These statements have been endorsed by the Chinese Society of Pediatric Endocrinology and Metabolism (CSPEM), European Society for Paediatric Endocrinology (ESPE), and Sociedad Latinoamericana de Endocrinología Pediátrica (SLEP), with the overarching goal of improving diagnostic clarity and decision-making with respect to treatment for children with ISS. Additionally, the Pediatric Endocrine Society (PES) affirms that this publication may be of educational value to their membership.

## Methods

### Selection of Steering Committee and Expert Panel Members

Two contributor groups were essential to the Delphi methodology: the Steering Committee (SC), which supported the development of the Delphi statements, and the Expert Panel (EP), which evaluated the statements for consensus. Members of both groups were selected based on their expertise and leadership in ISS, while also ensuring geographic diversity.

A structured process was used to ascertain expertise among identified pediatric endocrinologists who are key opinion leaders (KOLs) specializing in the management of ISS. Individuals were scored using a standardized matrix that evaluated 5 domains: (1) involvement in national or international endocrinology societies; (2) institutional affiliations, with emphasis on individuals holding leadership positions; (3) years of clinical and research experience; (4) level of engagement in scientific conferences, such as chairing sessions or delivering invited lectures; and (5) prior consulting experience, such as policy, guidance, or advisory roles.

In total, 43 experts were invited to participate in the process, 14 as members of the SC and 29 as members of the EP, with no overlap between the groups. The final SC consisted of 10 internationally recognized KOLs with extensive clinical and academic expertise in ISS, representing most regions included in the EP selection. The EP consisted of 22 highly influential KOLs representing the following major global regions of North America (*n* = 5), Europe (*n* = 9), Brazil (*n* = 1), China (*n* = 2), Israel (*n* = 2), and Japan (*n* = 3). Panelists had between 20 and 50 years of clinical experience.

### Drafting Initial Consensus Statements

To develop the initial set of consensus statements, a targeted non-systematic literature review was conducted across 5 core domains relevant to ISS management: (1) the definition and classification of ISS; (2) clinical and auxological diagnostic criteria; (3) recommended biochemical testing; (4) approaches to growth prediction and long-term follow-up; and (5) psychosocial considerations impacting evaluation and treatment decisions. Draft statements derived from this review were reviewed and finalized by the SC through web-based surveys and virtual meetings.

### The Modified Delphi Approach

A 3-round Delphi survey was administered to EP members using the Qualtrics online platform (Qualtrics, Provo, UT) between November 2025 and February 2026 (Fig. 1). In Round 1, all 22 participants evaluated each draft statement using a 6-point Likert scale ranging from “Strongly Agree” to “Strongly Disagree,” accompanied by qualitative descriptors to orient respondents toward expressing overall agreement or disagreement. Consensus was defined as at least 70% of panelists selecting any level of agreement (i.e., Somewhat Agree, Agree, or Strongly Agree), consistent with commonly used thresholds in Delphi methodology to indicate strong majority support while still allowing for some disagreement among panelists, and the process was not anonymous [10, 11]. Additionally, respondents provided feedback to highlight issues requiring clarification or modification, which was then reviewed by the SC and used to refine statements as needed. Round 2 involved re-evaluation by all 22 participants of any statements that did not reach consensus in Round 1 or that required substantial modification based on panelist feedback. Responses were single-blinded, and only visible to the SC and facilitator. The same 6-point scale and 70% consensus threshold were applied. In Round 3, all statements that had reached consensus in the previous rounds were compiled and distributed to all participants for final approval, confirming their agreement with the finalized set of consensus statements. There were no remaining statements that required additional modification or further consensus.

**Fig. 1.**
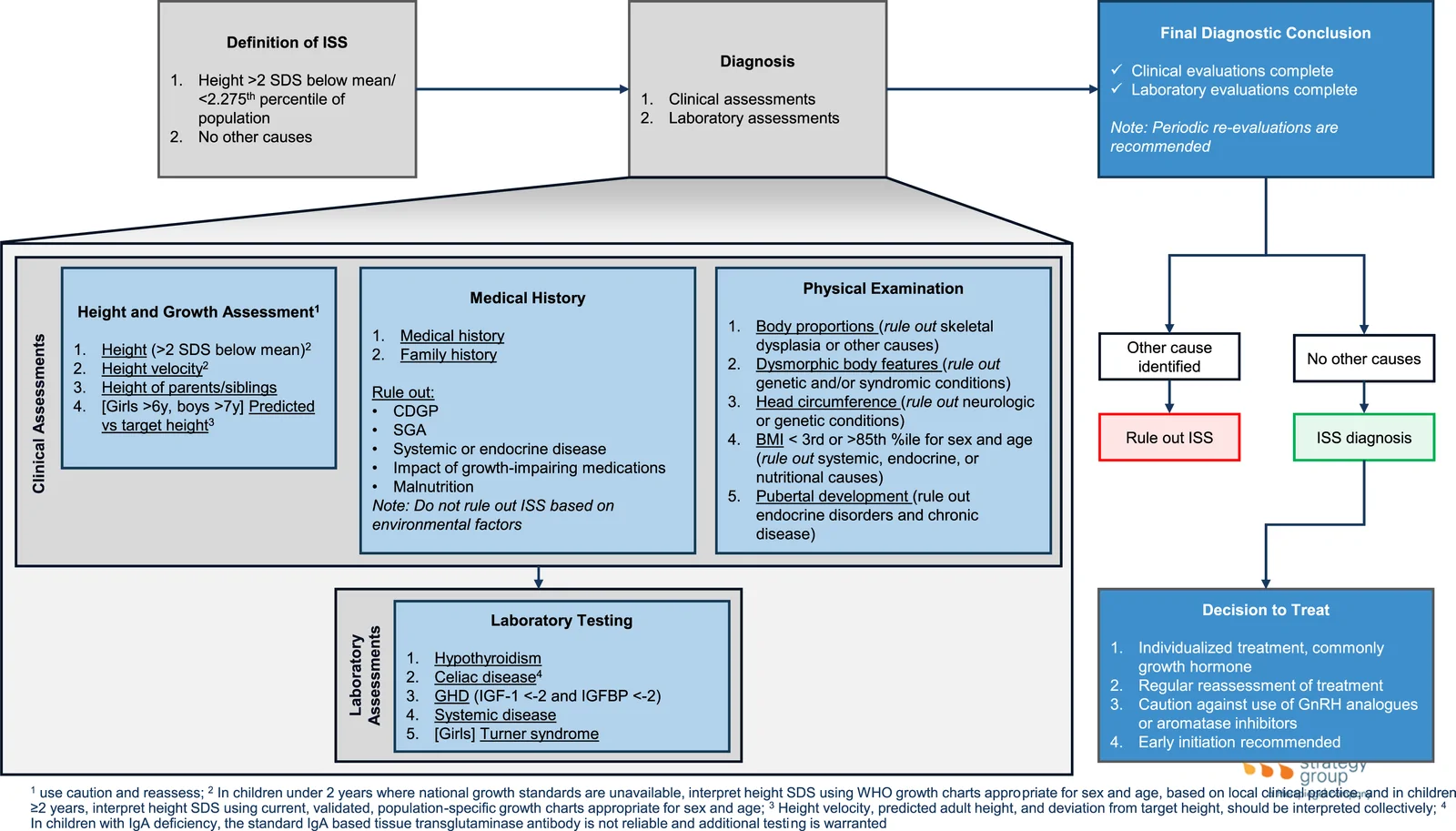
Algorithm for the diagnosis and clinical management of ISS. ^1^ Use caution and reassess; ^2^ In children under 2 years of age where national growth standards are unavailable, interpret height SDS using WHO growth charts appropriate for sex and age, based on local clinical practice, and in children ≥2 years of age, interpret height SDS using current, validated, population-specific growth charts appropriate for sex and age; ^3^ Height velocity, predicted adult height, and deviation from target height, should be interpreted collectively; ^4^ In children with IgA deficiency, the standard IgA-based tissue transglutaminase antibody is not reliable and additional testing is warranted. BMI, body mass index; CDGP, constitutional delay of growth and puberty; GHD, growth hormone deficiency; GnRH, gonadotropin-releasing hormone; HRQoL, health-related quality of life; IgA, immunoglobulin A; IGF-1, insulin-like growth factor 1; IGFBP-3, insulin-like growth factor–binding protein 3; ISS, idiopathic short stature; SDS, standard deviation score; SGA, small for gestational age; WHO, World Health Organization; y, years.

## Results

### Consensus Recommendations

Consensus recommendations were grouped by the following 4 categories: definition of ISS, diagnosis, burden of disease, and decision to treat. Percent consensus is provided for each recommendation, along with the Delphi round at which consensus was reached. A total of 44 statements reached consensus, with final agreement across all consensus statements ranging from 77% to 100%. A summary of the process, including the number of statements assessed per round, those achieving consensus, and the range of final agreement percentages, is presented in Table 1. An algorithm for the diagnosis and clinical management of ISS is shown in Figure 1.

**Table 1.** Summary of consensus process

Measure	Round 1	Round 2
Statements evaluated*	49	18
Statements achieving consensus	26	18
Range of consensus	77–100%	82–100%
Final number of statements included	--	44

*Statements from round 1 that did not reach consensus (i.e., <70% consensus; 23 statements) were modified or removed based on participant feedback prior to round 2 evaluations.

### Definition of ISS

Box 1. Recommendations on the definition of ISS

**Table:** 

1. ISS is defined as a condition in which an individual has a height more than a standard deviation score (SDS) of 2 below the mean for sex, age, and population, corresponding to below the 2.3 percentile, without identifiable underlying pathology (100%, Round 2).
2. ISS is a condition of exclusion and should only be considered after excluding known causes, including systemic diseases, endocrine disorders, nutritional deficiencies, skeletal dysplasia, and syndromic disorders (100%, Round 2).

### Additional Comments

The definition for ISS is based on clinical assessment complemented by laboratory and radiological investigations. With advances in genetic knowledge, it is now recognized that a substantial proportion of children classified as having ISS harbor an underlying monogenic cause of growth failure [12]. As access to genetic testing continues to expand, including the future incorporation of polygenic risk scores, a comprehensive redefinition of ISS is likely to occur [9].

### Diagnosis: Height and Growth Assessment

Box 2. Recommendations on diagnosis of ISS: Height and growth assessment

**Table:** 

1. In children under 2 years of age, measure recumbent length. In children ≥2 years of age in whom a standing height can be accurately obtained, measure standing height (95%, round 1).
2. In children ≥2 years, interpret height SDS using current, validated, population-specific growth charts appropriate for sex and age (95%, round 1).
3. In children under 2 years where national growth standards are unavailable, interpret height SDS using World Health Organization (WHO) growth charts appropriate for sex and age, based on local clinical practice (95%, round 2).
4. Evaluate height velocity using at least 2 height measurements over a minimum of 6 months (95%, Round 1).
5. If possible, measure the height of the child’s biological parents and siblings (100%, round 1).
6. For girls over 6 years and boys over 7 years, if possible, calculate the child’s predicted height SDS using bone age and growth prediction methods and compare it to their target height SDS (77%, round 1).
7. Caution should be exercised when predicting height based on bone age, and reassessment may be necessary during follow-up (86%, round 2).
8. Children with height more than 2 SDS below the mean for sex, age, and population or whose height is more than 2 SDS below their target height, should be evaluated for short stature (82%, round 1).
9. Height velocity, predicted adult height, and deviation from target height, should be interpreted collectively in the workup for ISS. While these parameters do not independently confirm or exclude ISS, abnormalities may warrant targeted evaluation for pathological causes (e.g., systemic, endocrine, nutritional) (91%, round 1).
10. The presence of short stature in one or both parents does not exclude the diagnosis of ISS in the child and may suggest the possibility of a genetic cause of short stature (91%, round 2).

### Additional Comments

When assessing height, current growth charts in children ≥2 years of age may include US Centers for Disease Control (CDC) curves or local reference data, while growth charts used in children <2 years of age may include standard World Health Organization (WHO) curves.

Height velocity can be assessed using established longitudinal reference standards and growth charts [13]. Widely used methods include the Tanner and Davies clinical longitudinal height and height velocity curves for North American children, the Bone Mineral Density in Childhood Study (BMDCS) annual height velocity reference ranges, and other height velocity curves accounting for timing of maturation [13, 14].

There are limitations to the use of height prediction methods in girls over 6 years and boys over 7 years, with predicted adult height being considered an adjunctive tool within the overall auxological assessment rather than a required determinant of ISS diagnosis or treatment eligibility. Predicted height (and predicted height SDS) derived from bone age-based methods can be imprecise, particularly in cases such as ISS where skeletal maturation is delayed or genetic causes of short stature where growth pattern may be abnormal; therefore, predicted height should be interpreted cautiously and should not be used as the sole criterion for diagnosis or treatment decisions.

Assessment of predicted adult height in children with ISS typically relies on established methods such as the Bayley-Pinneau approach, the Roche-Wainer-Thissen method, and the Khamis-Roche method [15–17]. However, bone age determination itself is subject to inter-rater variability and systematic differences depending on the method used, most commonly Greulich-Pyle versus Tanner-Whitehouse, which can affect predicted adult height estimates [18]. Beyond height prediction, bone age assessment plays an important role in the evaluation of ISS by helping to identify constitutional delay of growth and puberty (CDGP) and skeletal abnormalities suggestive of dysplasia.

### Diagnosis: Medical History

Box 3. Recommendations on diagnosis of ISS: Medical history

**Table:** 

1. Conduct a medical history evaluation to identify signs of underlying pathology. Include the child’s medical history (prenatal and perinatal history, history of chronic illnesses or infections, neurodevelopmental history, medication history), family medical history, and environmental factors and exposures (86%, round 2).
2. Family history may suggest constitutional delay of growth and puberty (CDGP), but this is a condition of exclusion confirmed only after delayed pubertal onset (95%, round 2).
3. Children with CDGP represent a distinct etiological group and should not be classified as having ISS (77%, round 1).
4. Children born small for gestational age (SGA) represent a distinct etiological group and should not be classified as having ISS (77%, round 1).
5. If systemic or endocrine disease is suspected, conduct targeted evaluations and follow-up to exclude these conditions (95%, round 1).
6. If growth-impairing medications are used, assess their impact before diagnosing ISS (95%, round 1).
7. If signs of malnutrition are present, conduct nutritional assessment and address deficiencies prior to ISS diagnosis (95%, round 1).
8. Environmental factors and exposures should be evaluated for their potential contribution to short stature, but these do not exclude a diagnosis of ISS. Clinical interpretation should consider these findings in the context of the full diagnostic evaluation (91%, round 1).

### Additional Comments

When conducting a medical history evaluation, the following data should be collected: (1) prenatal and perinatal history, including birth weight, birth length, head circumference, and gestational age; (2) history of chronic illnesses or infections, including gastrointestinal, renal, cardiac, endocrine, inflammatory; (3) neurodevelopment history, including hypotonia, developmental delay, autism spectrum disorder, or intellectual disability; and (4) medication history, including past or current use of growth-impairing agents, such as glucocorticoids, chemotherapy, or stimulant medications such as methylphenydate. Collection of the child’s family medical history should include consanguinity and pubertal timing in both parents, and heights of family members. Finally, environmental factors and exposures consist of (1) nutritional history, including dietary intake, feeding difficulties, and malnutrition; (2) low socioeconomic status; and (3) psychosocial factors, such as smoking or alcohol consumption during pregnancy and exposure to abuse or neglect. If an alternate explanation is found that explains the growth failure, such as use of a growth-impairing agent, the patient should not be diagnosed with ISS.

Although family history can suggest CDGP, it remains a diagnosis of exclusion that cannot be confirmed until delayed pubertal onset becomes evident. In prepubertal children with short stature, CDGP and ISS may not be distinguishable at initial evaluation. Delayed bone age by 2 or more years may support a diagnosis of CDGP but is not definitive. Therefore, such children should be considered as having provisional unexplained short stature and undergo longitudinal follow-up. Reassessment at expected pubertal milestones is suggested with delayed puberty, defined as the absence of breast development by age 13 years in girls or testicular enlargement by age 14 years in boys, further prompting reassessment of growth trajectory, skeletal maturation, and diagnostic classification. Children born SGA may have genetic etiologies which overlap with those that underlie ISS and should be considered when assessing for ISS (see International Genetic Guidelines for more details) [9, 12].

### Diagnosis: Physical Examination

Box 4. Recommendations on diagnosis of ISS: Physical examination

**Table:** 

1. Perform a physical examination to identify clinical signs of underlying pathology, including assessment of body proportions, dysmorphic body features, head circumference, body mass index (BMI), and pubertal development (95%, Round 2).
2. If there is evidence of body disproportion, evaluate for skeletal dysplasia or other causes of disproportionate short stature (100%, Round 2).
3. If significant dysmorphisms or major malformations are present, evaluate for genetic and/or syndromic conditions (95%, Round 1).
4. If head circumference is abnormal (microcephaly or macrocephaly), assess for neurologic or genetic conditions associated with short stature (95%, Round 1).
5. Abnormal BMI (<3rd or >85th percentile for sex and age) may suggest systemic, endocrine, or nutritional causes of short stature and warrants further evaluation (95%, Round 1).
6. Precocious puberty in a child with short stature warrants a thorough evaluation for endocrine disorders and chronic disease (91%, Round 2).

### Additional Comments

When performing a physical examination, the following metrics should be obtained: (1) body proportions using sitting height-to-height (SH:H) ratio and arm span, (2) dysmorphic body features involving the face, neck, trunk, and extremities, (3) head circumference, (4) BMI, and (5) pubertal development using Tanner staging [19]. Developmental and behavioral observations made during the clinical encounter may also provide supportive clues to an underlying syndromic or neurogenetic condition and should be integrated with medical history when determining the need for further evaluation.

Interpretation of any identified body disproportion should be assessed based on SH:H SDS ratio and arm span minus height SDS. If either measurement is >+2 SDS or < −2 SDS from the mean for sex and age, based on population-specific reference charts, evaluate for skeletal dysplasia or other causes of disproportionate short stature. In patients with evidence of disproportion, a skeletal survey may provide evidence for the diagnosis of a distinct skeletal dysplasia.

### Diagnosis: Laboratory Testing

Box 5. Recommendations on diagnosis: Laboratory testing

**Table:** 

1. If clinical evaluation reveals abnormalities, perform targeted laboratory testing based on suspected etiology (100%, round 1).
2. If no abnormalities are found upon clinical evaluation, perform screening laboratory tests to exclude common causes of short stature before diagnosing ISS. Include:
a. Thyroid function: Measure thyroid-stimulating hormone (TSH) and free thyroxine (free T4)
b. Celiac disease: Perform serologic screening with tissue transglutaminase IgA (tTG-IgA) and total IgA.
c. GH deficiency (GHD): Measure serum IGF-1; In children <3 years, IGF-binding protein 3 (IGFBP-3) may be more informative.
d. Systemic diseases: Measure systemic disease markers based on clinical suspicion (82%, round 2).
3. If thyroid function is abnormal, evaluate for hypothyroidism (100%, round 1).
4. If celiac serology is positive, evaluate for celiac disease. In children with IgA deficiency, the standard IgA-based tissue transglutaminase antibody is not reliable and additional testing is warranted (100%, round 1).
5. For IGF-1, a value below −2 SDS is highly suggestive of GHD, but normal values do not exclude milder forms. For IGFBP-3, a value below −2 SDS is also considered abnormal, with higher specificity in early childhood. Combined low IGF-1 and IGFBP-3 values increase specificity for GH deficiency/resistance (82%, round 2).
6. If biochemical screening reveals abnormalities, investigate for systemic disease (100%, round 1).
7. The diagnosis of ISS is reached after exclusion of identifiable causes; advances in genetic testing may subsequently reveal an underlying etiology (95%, round 2).
8. All girls with unexplained short stature should undergo analysis to rule out Turner syndrome, regardless of presence or absence of clinical features (86%, round 2).

### Additional Comments

Some experts noted that the yield of routine diagnostic laboratory testing may be low in asymptomatic children with short stature who have normal growth velocity and no concerning clinical findings. Therefore, although baseline testing was supported by the majority of panelists, the extent of evaluation should be interpreted in the context of auxology, growth velocity, history, and physical examination [20]. When performing screening laboratory testing, systemic disease should be investigated as appropriate and consists of performing general biochemical screening, which may include complete blood count, erythrocyte sedimentation rate, C-reactive protein, and renal, liver, thyroid antibodies and free tri-iodothyronine (FT3), and metabolic function tests. Radiological studies may be helpful in patients with suspected skeletal dysplasia.

ISS is typically diagnosed when stimulated GH concentrations exceed the deficiency threshold (commonly a peak GH ≥7.5–10 μg/L on two tests, though cutoffs vary by assay and region) and when IGF-1 and IGFBP-3 are within normal range [21]. The use of GH stimulation testing in ISS remains controversial, however, with some experts arguing that it may be unnecessary in otherwise healthy children with adequate growth velocity [22].

Of note, when assessing for GHD, IGFBP-3 is the preferred metric in children <3 years of age as it is more specific for GHD than IGF-1. Potential differential diagnoses for low IGF-1/IGFBP-3 concentrations, beyond GH deficiency or resistance include acid-labile subunit deficiency [21], malnutrition, anorexia nervosa, type 1 diabetes mellitus, chronic inflammatory diseases (e.g., Crohn’s disease or juvenile chronic arthritis) [23], and celiac disease.

In children with IgA deficiency, the recommendation that additional testing be conducted is aligned with European Society for Paediatric Gastroenterology, Hepatology and Nutrition (ESPGHAN) recommendations, according to which screening for celiac disease should be performed using tissue transglutaminase IgA antibodies (tTG-IgA) with concurrent measurement of total serum IgA. When there is low or absent total IgA (i.e., IgA deficiency), tTG-IgA is unreliable. Instead, IgG-based serologic tests, such as tTG-IgG or deamidated gliadin peptide IgG, should be used [24].

When testing for Turner syndrome in girls with unexplained short stature, the standard approach is peripheral blood karyotyping, which can detect 45,X monosomy, mosaicism (e.g., 45,X/46,XX), and structural abnormalities of the X-chromosome [25]. However, if the karyotype is normal but clinical suspicion remains based on physical characteristics, high-resolution karyotyping or fluorescence in situ hybridization can be used to improve detection of low-level mosaicism or cryptic X-chromosome abnormalities [25]. In some centers, chromosomal microarrays or whole genome sequencing have replaced karyotyping as the screening test for Turner syndrome [26, 27]. Genetic testing is not required to assign the initial clinical label of ISS in every child; however, it should be considered when phenotypic features, family history, neurodevelopmental findings, disproportion, or local diagnostic pathways raise suspicion for an underlying monogenic or syndromic cause of short stature, in accordance with recently published international guidance.

### Diagnosis: Final Diagnostic Conclusion

Box 6. Recommendations on diagnosis of ISS: Final diagnostic conclusion

**Table:** 

1. If all recommended clinical and laboratory evaluations have been completed and no pathological cause of short stature has been identified, the child may be classified as having ISS (91%, round 2).
2. Children diagnosed with ISS should be periodically re-evaluated to monitor growth progression and assess for emerging clinical signs suggestive of an underlying pathological cause (100%, round 1).

### Additional Comments

ISS is a clinical diagnosis of exclusion that may be revised over time as new clinical features emerge or additional investigations become available. Periodic reassessment should include review of growth trajectory, pubertal progression, interval medical history, and any new findings suggestive of endocrine, systemic, skeletal, or genetic disease.

### Burden of Disease

Box 7. Recommendations on burden of disease of ISS

**Table:** 

1. Some children and adolescents with short stature may experience reduced HRQoL (91%, round 2).
2. Some parents of children with short stature may experience increased psychological and emotional burdens due to caregiving responsibilities, psychosocial stress, and concerns about their child’s future health, development, and social integration (95%, round 1).
3. The impact of short stature on quality of life can vary widely between individuals. While some children and families experience psychosocial challenges, others report minimal or no impact (100%, round 1).

### Additional Comments

Specific domains of HRQoL that may be affected in children and adolescents with ISS include emotional challenges, social difficulties, and internalizing problems. These impacts have been assessed using validated tools such as the (Children Quality of Life Questionnaire) KINDL questionnaire, the Pediatric Quality of Life Inventory (PedsQL), the Quality of Life in Short Stature Youth (QOLISSY) questionnaire, and the Youth Quality of Life-Short Stature module (YQOL-S). The magnitude of HRQoL impairment appears to vary according to moderating factors including the degree of short stature, age and developmental stage, cultural context, family support, and the presence of comorbid conditions. However, the overall strength and consistency of the evidence remain variable, with heterogeneous study designs and mixed findings across populations [5, 28, 29].

### Decision to Treat

Box 8. Recommendations on decision to treat ISS

**Table:** 

1. For children diagnosed with ISS after exclusion of pathological causes, growth-promoting treatment, most commonly with GH, should be individualized and pursued through a shared decision-making process. This approach should integrate clinical expertise, the child’s and family's values and preferences, and a balanced discussion of the potential risks, benefits, costs, and expected outcomes. Treatment decisions should be regularly reassessed based on response and evolving goals (100%, round 1).
2. Some children with ISS may spontaneously reach an adult height within the normal range for their population or target height without treatment (91%, round 1).
3. Puberty modulation with the use of gonadotropin-releasing hormone (GnRH) analogs or aromatase inhibitors should not be routinely proposed in children with ISS due to limited information on benefits regarding adult height and safety (95%, round 1).
4. GH treatment may improve quality of life in short-statured children and their parents (82%, round 2).
5. Earlier initiation of GH treatment, after thorough evaluation, is associated with better height outcomes in some cohorts (91%, round 2).

### Additional Comments

Registry data from the Kabi/Pfizer International Growth Database (KIGS) database, consisting of 4,685 children with ISS, demonstrated that younger, prepubertal age at GH start was linked to greater overall height gain and adult height SDS, with treatment response declining with increasing age at initiation [30]. Additionally, observational studies have shown that patients who begin GH treatment at a younger age and continue it for longer durations achieve larger improvements in height SDS compared with those who start later, supporting the potential benefit of early therapy in maximizing height outcomes [31]. However, given the variability in treatment response among children with ISS, decisions regarding GH therapy should be individualized and based on careful patient selection.

Predicted adult height based on bone age should be considered when assessing the potential benefit of GH treatment in children with ISS. In addition, pubertal status may be an important consideration for treatment initiation. The evidence supporting meaningful height gains with initiation of GH therapy after the onset of puberty, particularly in children already experiencing a pubertal growth spurt, is limited. Given the substantial cost of treatment, the likelihood of benefit based on both predicted adult height and stage of pubertal development should be incorporated into treatment decision-making.

## Discussion

The international diagnostic consensus statements presented here represent, to our knowledge, the first ISS-focused recommendations developed through a structured Delphi process with broad international expert representation, with the intent of providing practical guidance for identifying children and adolescents with ISS and supporting clinical decision-making regarding management.

Importantly, this consensus initiative was deliberately focused on the clinical diagnosis of ISS, with genetic evaluation being considered beyond the scope of this process. Recent international recommendations on genetic testing provide a structured approach to evaluating children with short stature, highlighting clinical and diagnostic features that increase the likelihood of an underlying genetic etiology, thus allowing the statements presented here to remain tightly aligned with clinical diagnostic workflows and decisions encountered in everyday practice [9]. Genetic testing should, however, be considered when clinically necessary, taking into account phenotypic features and local availability.

Debate persists regarding the burden of disease in individuals with ISS. Some studies report that children with ISS have poorer HRQoL and greater psychosocial burden than children of average height, including behavioral and emotional challenges [5, 28, 32]. Other studies have not observed such associations, underscoring uncertainty about the condition’s clinical significance [29, 33]. Similarly, the extent to which ISS affects the quality of life of parents is debated, with some studies suggesting that parents experience increased concern, stress, or social worry related to their child’s growth, while other research found minimal psychosocial impact [34]. Given the heterogeneity in research findings regarding HRQoL impact, the EP concluded that while not all children with ISS experience psychosocial burden, short stature has the potential to negatively affect HRQoL in a meaningful subset of patients. Individual assessment of psychosocial functioning should therefore be part of the comprehensive evaluation, recognizing that the presence and severity of HRQoL impairment may influence treatment decisions.

The primary impact of these recommendations is improved consistency in identifying children who meet clinical criteria for ISS, thereby reducing diagnostic ambiguity and supporting more appropriate counseling and treatment planning. By promoting a standardized diagnostic pathway, these statements aim to help clinicians systematically rule out other causes of growth failure (e.g., chronic inflammatory disease, endocrine disorders, or SGA), which is critical to ensuring that children receive appropriate management strategies aligned with ISS.

Regulatory and access barriers remain important implementation challenges for GH therapy in ISS. While recombinant human GH was approved for ISS by the US Food and Drug Administration in 2003, it has not been authorized for this indication by the European Medicines Agency and remains off-label in many countries, contributing to variability in prescribing practices. The consensus statements provided here may facilitate policy discussions in such settings, including regions where GH for ISS is not yet approved.

This modified Delphi process had limitations that should be considered. First, the consensus statements reflect expert opinion synthesized from clinical experience and targeted evidence review rather than a standard systematic literature review and may therefore be influenced by both the composition of the panel and the limited literature available for inclusion. Second, although many global regions were represented in the EP, regions such as Africa and South Asia were not included. Finally, these statements provide recommendations rather than formal guidelines, as a formal guideline-development process (e.g., Grading of Recommendations Assessment, Development and Evaluation), including a systematic review methodology and explicit grading of evidence and recommendations, was not undertaken.

Future research should therefore prioritize generating clinical evidence to inform treatment-focused guidance, including clearer criteria for patient selection, clinically meaningful outcomes, and longer-term safety and benefit-risk assessment across diverse populations. In particular, long-term outcome studies following treated versus untreated individuals with ISS into adulthood, identification of biomarkers predictive of GH treatment response, changes in psychological burden, cost-effectiveness analyses, and studies clarifying genetic etiologies and their therapeutic implications are needed. Comparative studies evaluating different GH dosing regimens and treatment durations, as well as randomized controlled trials of GH in ISS are expected to help address this gap. Ultimately, we anticipate that convergence of standardized diagnostic pathways and stronger treatment evidence could support the development of formal international treatment guidelines and, over time, may facilitate more consistent access and reimbursement policies for appropriately selected patients globally.

To conclude, these International Consensus Statements provide a practical, harmonized framework to strengthen the identification and clinical management of ISS. The adoption of these statements may help standardize clinical practice and accelerate progress toward broader alignment in treatment decisions across regions, thereby promoting equity and improving the lives of individuals affected by this condition.

## Acknowledgments

Medical writing support was provided by Breni Sharma, PhD and Lauren Todd, PhD, CMPP, and study conduct support was provided by Edward Cruise, MSc, from Acumetis. The authors thank all participants in the Delphi process for their valuable contributions to this study.

## Statement of Ethics

This modified Delphi process involved expert participants providing professional opinions and did not include vulnerable populations or sensitive personal data. As such, ethical approval was not required for this study in accordance with local/national guidelines. Written informed consent to participate was not directly obtained but inferred by completion of the survey.

## Conflict of Interest Statement

A.L. has received honoraria from Novo Nordisk, Pfizer, Merck, Sandoz, Ascendis Pharma A/S; S.C. has received honoraria from Novo Nordisk, Sanofi and Sandoz; M.T.D. has served as an advisory board member for Novo Nordisk, Pfizer, Rhythm, has received consulting fees from Novo Nordisk, Sandoz, and has served as a speaker at meetings funded by Novo Nordisk, Pfizer, Neurocrine, Sandoz; A.A.L.J. has received speaker fees from Novo Nordisk, Merck, Pfizer, a research grant from BioMarin, and consulting fees from Novo Nordisk; Y.L. has received honoraria/speaker fees from Lilly, Medison Pharma Ltd, Novo Nordisk, Pfizer, and Sanofi, has served as consultant/on advisory boards for Lilly, Medison Pharma Ltd, Novo Nordisk, and Sanofi, has received research grants/funding from Medison Pharma Ltd, Novo Nordisk, Pfizer, and Sanofi, and has received travel/accommodation support from Amryt Pharma, Chiesi Farmaceutici, Novo Nordisk, Sanofi; X.L. is an investigator and consultant for GenSci, Amoytop, Anke Bio, Visen, Novo Nordisk, Takeda, Lumos, Sanofi, Ipsen and Medtronic; M.M. has participated as a data safety monitoring/advisory board member and received consulting fees and honoraria from Pfizer, Novo Nordisk, Merck, Sandoz, Ascendis Pharma A/S and BioMarin; B.S.M. is a consultant for Amgen, Ascendis Pharma, BioMarin, Eton Pharmaceuticals, Novo Nordisk, Pfizer, Soleno, and Tolmar and has received research support from Aardvark Therapeutics, Alexion, Abbvie, Aeterna Zentaris, Foresee, Lumos Pharma, Novo Nordisk, OPKO Health, and Pfizer; R.R. is a consultant for Novo Nordisk; P.F.C.S. has received speaker and consulting fees and travel support to scientific meetings from BioMarin, Novo Nordisk, and Pfizer; and A.D. has served as a consultant for Novo Nordisk, Pfizer, Ascendis, Sandoz, Tyra, Eton, QED, and BioMarin and has grant funding from BioMarin and Pfizer. S.C. and A.D. were members of the journal’s Editorial Board at the time of submission. H.M. has nothing to disclose.

## Funding Sources

This study and manuscript development was financially supported by Novo Nordisk Healthcare AG and Ascendis Pharma A/S and conducted by Acumetis. Novo Nordisk Healthcare AG and Ascendis Pharma A/S had no role in the study design; collection, analysis, and interpretation of data; writing and approving the publication; or decision to submit the article for publication. Novo Nordisk Healthcare AG and Ascendis Pharma A/S performed medical accuracy reviews of the first and final drafts. All authors maintained control over the final content and contributed to content development. No author received payment or other compensation related to the writing of the manuscript.

## Author Contributions

A.D. contributed to the conceptualization and methodology of the study. A.L., S.C., M.T.D., A.A.L.J., Y.L. X.L., M.M., B.S.M., H.M., R.R., P.F.C.S., and A.D. participated in the Delphi expert consensus exercise and contributed to the drafting and review of the manuscript. S.C. and A.D. contributed to supervision of the study.

## Data Availability

The data generated and analyzed during this study are not publicly available due to the potential identifiability of participants but are available from the corresponding author on reasonable request.
